# A longitudinal study on social support, social participation, and older Europeans’ Quality of life

**DOI:** 10.1016/j.ssmph.2021.100747

**Published:** 2021-02-03

**Authors:** Septi Kurnia Lestari, Xavier de Luna, Malin Eriksson, Gunnar Malmberg, Nawi Ng

**Affiliations:** a Department of Epidemiology and Global Health, Umeå University, Umeå, 90187, Sweden; b Centre for Demographic and Ageing Research, Umeå University, Umeå, 90187, Sweden; c Umeå School of Business, Economics and Statistics, Umeå University, Umeå, 90187, Sweden; d Department of Social Work, Umeå University, Umeå, 90187, Sweden; e Department of Geography, Umeå University, Umeå, 90187, Sweden; f School of Public Health and Community Medicine, Institute of Medicine, Sahlgrenska Academy, University of Gothenburg, Gothenburg, 40530, Sweden

**Keywords:** Social support, Social participation, Quality of life, Older population, Panel data

## Abstract

The association between quality of life (QoL) and social relationships is well established. This paper further analyses whether and how participation in social activities as well as providing and receiving social support, independently, are associated with QoL among the older population in 16 European countries. QoL was measured using the CASP-12 scale. The baseline data came from Wave 6 and the outcome from Wave 7 of the Survey of Health, Ageing and Retirement in Europe (SHARE). The associations of interest were analysed using multivariable linear regression. The effect of possible non-ignorable dropout was tested. Then, doubly robust estimation and sensitivity analyses for unobserved confounding were performed to evaluate the possible causal interpretation of the associations found. Our findings show that participation in at least one of the socially productive activities was positively associated with QoL at two-year follow-up (Average Causal Effect, ACE: 0.474; 95%CI: 0.361, 0.587). The association was stronger among women, people aged 75+, and those in the Southern European region. Providing social support had a positive association with QoL, but only among people aged 75+ (ACE: 0.410; 95%CI: 0.031, 0.789). Conversely, receiving social support had a negative association (ACE: -0.321; 95%CI: -0.448, -0.195) with QoL, especially for men, people aged 75+, and those in Eastern European countries. Sensitivity analyses for unobserved confounders showed that the associations found cannot be attributed to causal effects.

## Highlights

• Association between exposure and outcome was tested using Doubly-robust analysis.
• Social participation was positively associated with older adults' QoL.
• Receiving social support was negatively associated with older adults' QoL.
• Providing social support was positively associated with the QoL of people age 75+ only.
• The associations observed were sensitive to the unobserved confounders.

## 1 Introduction

Population ageing is often regarded as a negative phenomenon that is characterised by worsened health status and poorer social life among older people. However, this is not always the case; in recent decades, most parts of the world have seen increasing healthy life expectancies (Grundy & Murphy, 2018, pp. 11-18; Mathers et al., 2001). Moreover, measuring the outcome of ageing solely in terms of physical health and functioning may be inadequate, as the goal of healthy ageing is not physical health but well-being (World Health Organization, 2015). Consequently, well-being and quality of life (QoL) measures are increasingly used to assess the health outcomes of old age (Bond & Corner, 2004; Hyde, Wiggins, Higgs, & Blane, 2003; World Health Organization, 2015). Furthermore, QoL has been used as an endpoint in the evaluation of public policy (e.g. social care) implementation, especially in Europe (Eurofound, 2013).

QoL is a complex concept that is amorphous, multi-layered, dynamic, and related to a range of components that interact. These components are both objective and subjective, involve multiple domains (environmental, social, health, and psychological), on the individual and the societal level, and can be positive and negative (Brown et al., 2004; Fernández-Ballesteros, 2011). The most common determinants of QoL are health status and social relationships (Bowling et al., 2003; Netuveli, Wiggins, Hildon, Montgomery, & Blane, 2006; Ward et al., 2019; Zaninotto et al., 2009). Indicators of poor mental and physical health – such as depression, longstanding illness, functional limitation, difficulties in activities of daily living (ADL) or instrumental activities of daily living (IADL), and poor self-rated health – were found to be negatively associated with QoL in older adults (Netuveli, Wiggins, Hildon, Montgomery, & Blane, 2006; Ward et al., 2019; Zaninotto et al., 2009). Male gender and socioeconomic factors, such as poor perceived financial situation, being unemployed or unable to work, low economic status, and low education level, also had negative associations with QoL (Netuveli, Wiggins, Hildon, Montgomery, & Blane, 2006; Ward et al., 2019; Zaninotto et al., 2009).

On the other hand, studies have shown that social relationships have mixed associations with QoL. Social relationships can be seen as resources for older people to fulfil their needs, in turn influencing their QoL. However, not all relationships are positive; some can be the source of stress, conflict, and disappointment (Dykstra, 2015, pp. 88-93). For instance, among European older adults, trusting relationships with family and friends, frequent contact with friends (Netuveli, Wiggins, Hildon, Montgomery, & Blane, 2006), and higher social network score (Ward et al., 2019) have been shown to be positively associated with QoL. However, living with someone, frequent contact with children and family (Netuveli, Wiggins, Hildon, Montgomery, & Blane, 2006), living with a partner, and loneliness were negatively associated with QoL (Ward et al., 2019). In contrast, data from the Survey of Health, Ageing and Retirement in Europe (SHARE) showed that among Europeans aged 60–79, having a partner and contact with children were associated with higher QoL (Litwin & Stoeckel, 2013).

Similarly, perceived support (the perception or belief that support is available when needed) has been consistently related to higher QoL (Turner & Turner, 2013, pp. 341-356; Zaninotto et al., 2009). Giving and receiving support, on the other hand, can be both negatively and positively associated with well-being. Providing informal help to others is associated with higher QoL (Litwin & Stoeckel, 2013; Potočnik & Sonnentag, 2013; Wahrendorf et al., 2006) and lower depression (Inagaki & Eisenberger, 2016; Piferi & Lawler, 2006). However, providing personal care is associated with poorer mental health (Hiel et al., 2015; Pinquart & Sörensen, 2006) and lower QoL (Litwin & Stoeckel, 2013; Netuveli, Wiggins, Hildon, Montgomery, & Blane, 2006; Wahrendorf et al., 2006). Receiving support has also been linked to both better (Gariépy et al., 2016; Siedlecki et al., 2014) and worse (Gleason et al., 2008; Litwin & Stoeckel, 2013; Reinhardt et al., 2006) well-being. A large survey on social support and older adults’ autonomy in Norway, England, Germany, Spain, and Israel also reported that received support from families and public services was negatively related to QoL (Tesch-Römer, Motel-Klingebiel, & von Kondratowitz, 2003, pp. 275-280). On the other hand, participation in social activities tends to have a positive association with QoL in older adults (Cattan et al., 2011; Litwin & Stoeckel, 2013; Netuveli, Wiggins, Hildon, Montgomery, & Blane, 2006; Newman et al., 2014; Omorou et al., 2013; Potočnik & Sonnentag, 2013; Siegrist & Wahrendorf, 2009; Wahrendorf et al., 2006; Wahrendorf & Siegrist, 2010; Ward et al., 2019).

Furthermore, studies have reported the heterogeneity of the effects of social relationship aspects on QoL. The association between social relationships and QoL in older adults can vary across age group, gender, and country of residence (Ioannidi & Mestheneos, 2013, pp. 151-162; Litwin & Stoeckel, 2013; Netuveli, Wiggins, Hildon, Montgomery, & Blane, 2006; Rollero et al., 2014). The variation by age may be explained by how different age groups rank the determinants of QoL (health and social relationships) (Bond & Corner, 2004). It has been demonstrated that the older group tends to perceive their health as the most important factor for their QoL (Bond & Corner, 2004). This is reflected in the finding by Litwin and Stoeckel (2013) that the effect of social relationships on QoL was less pronounced among people aged 80+ compared to those aged 60–79.

As for gender difference, social relationship indicators such as being unemployed due to taking care of one's family or home, as well as more frequent contact with children and family, have been reported to reduce older women's QoL but not older men's. At more advanced ages, having a partner has been associated with higher QoL in older men but not in older women (Zaninotto et al., 2009). A study on people aged 75+ in Germany reported a moderating effect of gender on the association between perceived social support and health-related QoL, with a positive effect of social support observed only among men (Hajek et al., 2016). Among Italian adults, however, social support had a stronger association with QoL among women (Rollero et al., 2014).

The gender difference in the effect of social relationships on QoL might be explained by cultural constructions of femininities and masculinities. Due to cultural gender beliefs, men and women are expected to behave differently in virtually all social arenas, including family and other social relations (Courtenay, 2000). Connell (2002) describes how, for instance, the structure of productive relations in modern Western society defines the domestic (social-supportive) sphere as the woman's world, based on cultural beliefs regarding masculine and feminine tasks, while the economic sphere is defined as the man's world. These masculine, as opposed to feminine, qualities may cause men to perceive receiving support as more harmful to their esteem than women do (esteem enhancement theory).

In addition, men and women typically provide different types of support. Emotional support and personal care are commonly provided by women, while men are more likely to help with paperwork and odd jobs in and around the house (Dykstra, 2015, pp. 88-93; Liebler & Sandefur, 2002). Considering the potential negative effect of providing personal care on QoL, women who mostly provide personal care may have lower QoL than men who provide other types of support.

Furthermore, contextual factors such as culture, welfare policies, and social change are the likely source of heterogeneity in the association between social support and QoL (Berkman & Krishna, 2014, pp. 273–319). The different cultures and family structures, and the availability of public services for older adults, may lead to variation in the frequency and type of support exchanged (Schmid et al., 2012). Services may also be more acceptable in one society than in another (Tesch-Römer, Motel-Klingebiel, & von Kondratowitz, 2003, pp. 275-280). For instance, in countries with more traditional family values, where family is expected to be the main provider of care for its members, receiving care or help from friends or public services may be valued negatively by support recipients (Polverini & Lamura, 2005, pp. 179-200). Also, being the sole care provider with limited, if any, support from the public services may be perceived as a burden. On the other hand, in countries where it is culturally acceptable for public services to take on a more intensive caring role, the share of support provided voluntarily by family members may be more positively valued.

Similarly, certain types of social activity may be preferred more in one society than in others. Non-kin-based social activities may be less common and less desired in familistic countries in Eastern and Southern Europe, while the opposite is true for countries with individualistic values, such as those in Northern Europe (Mair, 2013, pp. 61–81). As social participation and giving or receiving support may be valued differently in different societies, their respective associations with QoL may also differ between societies.

Both active participation in social activity and social support are important aspects of active ageing policy in Europe (Foster & Walker, 2015; World Health Organization, 2012). However, the evidence for the effect of social support and social participation on QoL has mostly originated from cross-sectional studies (Cattan et al., 2011; Liang, Krause, & Bennett, 2001; Turner & Turner, 2013, pp. 341-356). As the time sequence between social relationships and QoL cannot be ascertained in a cross-sectional design, reverse causation cannot be ruled out. Additionally, studies in this field more often focus on intergenerational support, with older people as the recipients of the support (Tesch-Römer, Motel-Klingebiel, & von Kondratowitz, 2003, pp. 275-280). Studies assessing the effect of providing support with older adults as the providers are limited, despite the fact that older adults often continue to provide emotional and instrumental support for their family until very late in life. This role reverses only after they begin having health difficulties (Dykstra, 2015, pp. 88-93). Furthermore, country comparison analysis is important for determining whether social relationships exhibit the same effect on QoL in different societies.

Against this background, this study used panel data from 16 European countries to address the following questions: 1) Are participation in socially productive activities, receiving social support, and providing social support independently associated with quality of life among older Europeans? 2) Do these associations differ by gender, age group, and country group based on their welfare models? 3) Can we make a causal interpretation of the association observed?

## 2 Methods

### 2.1 Data source

We used panel data from Waves 6 and 7 of the Survey of Health, Ageing and Retirement in Europe (SHARE) (release 7-0-0). SHARE, a cross-national longitudinal survey covering most European countries and Israel, collects data on a wide range of ageing-related indicators including health, socioeconomic status, and social and family relationships. The first wave of SHARE was implemented in eleven countries during the years 2004–2005. Older adults aged 50+ who were not institutionalised and their partners (irrespective of their age) were eligible to participate. Follow-up rounds of data collection were conducted approximately every two years. In 2017, 28 countries implemented Wave 7 of SHARE. All respondents who participated in any previous waves of SHARE are part of the longitudinal sample. Details on survey methods and instruments are available elsewhere (Börsch-Supan et al., 2013).

### 2.2 Study sample

Seventeen European countries (Austria, Belgium, Croatia, Czech Republic, Denmark, Estonia, France, Germany, Greece, Italy, Luxembourg, Poland, Portugal, Spain, Slovenia, Sweden, and Switzerland) completed SHARE Waves 6 and 7. However, Portugal was excluded from this study as at the time of analysis its Wave 7 data had not been released. A total of 64,478 individuals from the other 16 countries participated in SHARE Wave 6. A total of 865 respondents younger than 50 years old, and 721 respondents who had at some point resided in a nursing home, were excluded from this study. Of the rest, 22% had missing data in at least one of the baseline variables as measured in Wave 6, and were therefore excluded; this resulted in an analytical sample size in the baseline of 49,214. Around 77% (n = 37,908) of these respondents had data on the outcome variable measured in Wave 7.

### 2.3 Measurements

#### 2.3.1 Quality of life

SHARE uses CASP-12, an abridged version of CASP-19, to measure QoL. Both versions of CASP exhibited good psychometric properties with good internal reliability and concurrent validity (Hyde, Wiggins, Higgs, & Blane, 2003; von dem Knesebeck, Hyde, Higgs, Kupfer, & Siegrist, 2008, pp. 199–234). The CASP scale was developed based on the ‘need satisfaction’ theory for measuring QoL in older adults. According to this theory, human beings share a common set of needs, and the extent to which these needs are fulfilled translates into their level of subjective well-being (Diener & Lucas, 2000). The CASP scale measures the degree of fulfilment of four domains of needs: **C**ontrol, **A**utonomy, **S**elf-realisation, and **P**leasure (Hyde, Wiggins, Higgs, & Blane, 2003) (see Table A1 in the appendix). Each of the items is assessed on a four-point Likert scale (‘often’, ‘sometimes’, ‘rarely’, ‘never’). The CASP score is the sum of points from these 12 items, ranging from 12 to 48 whereby a higher score indicates a higher subjective QoL. A high QoL (CASP score) can be achieved when one is free from undue interference (autonomy), able to intervene in one's environment (control), and involved in an active and reflexive process of self-realisation through activities that bring happiness (pleasure) (Hyde, Wiggins, Higgs, & Blane, 2003). In the present study, QoL was measured in Wave 6 as the baseline level and in Wave 7 as the main outcome.

#### 2.3.2 Social participation

Respondents were asked to identify at least one activity, from a list of activities, that they had engaged in during the preceding 12 months. To indicate participation in social activities (one of the three exposures of interest), we derived a binary variable indicating whether a respondent took part in at least one of the four socially productive activities assessed in this study; i.e., voluntary/charity work, educational/training course, sport/social club, and political/community organisation.

#### 2.3.3 Providing and receiving social support

Two of the three main exposures of interest in this study – i.e., receiving support and providing social support – were each assessed using a single item measure. Respondents were asked whether during the last 12 months they had provided any instrumental support (i.e., personal care, practical household help, or paperwork-related help) to friends, neighbours, or family outside their household. A similar question was used to assess receiving support.

#### 2.3.4 Other social relationship measures

Social network size was measured by asking respondents to list up to six persons (family members, friends, neighbours, or other acquaintances) with whom they had discussed important things during the preceding 12 months, and one additional person who was important to them for any reason.

The average frequency of contact with social network members was categorised as ‘never’, ‘occasionally’ (every 2 weeks, 1/month, <1/month), and ‘frequently’ (daily, >1/week, 1/week). Household size and number of living children were recorded as reported by respondents. Marital status was grouped as ‘with partner’ (married/had registered partner) and ‘without partner’ (never married/divorced/widowed).

#### 2.3.5 Sociodemographic measures

Age was calculated based on date of birth and interview date. Gender included men and women. Education level was recorded according to the International Standard Classification of Education (ISCED) 2011, which ranges from 0 to 6. We categorised education level as ‘low’ (ISCED 0, 1, and 2), ‘middle’ (ISCED 3 – Upper secondary education and 4 – Post-secondary non-tertiary), and ‘high’ (ISCED 5 and 6). Employment status was classified as ‘employed’ (which included ‘self-employed’), ‘retired’, ‘unable to work’ (permanently sick or disabled), and ‘not employed’ (which included ‘unemployed’ and ‘homemaker’). Perceived household economic status was measured according to whether the ‘household was able to make ends meet’*,* with responses grouped as ‘with difficulty’ (‘with great difficulty’ or ‘with some difficulty’) or ‘easily’ (‘fairly easily’ or ‘easily’).

The 16 countries in this study were grouped into four regions representing their welfare regime types (Eurofound, 2013; Niedzwiedz et al., 2014): Social Democratic/Nordic (Sweden and Denmark), Corporatist/Central (Austria, Belgium, France, Germany, Luxemburg, Switzerland), Post-Socialist/Eastern (Croatia, Czech Republic, Estonia, Poland, and Slovenia), and Southern European/Mediterranean (Spain, Italy, and Greece).

#### 2.3.6 Other health-related measures

Cognitive function was measured based on the assessment of three cognitive tasks: verbal fluency, immediate word recall, and delayed word recall. In the verbal fluency test, respondents were asked to say as many words from the animal category as they could in 60 s. The fluency score ranged from 0 to 100. The fluency test reflects one's executive function, while the word recall test reflects memory performance. In the word recall test, the interviewer read a list of ten words once. Then respondents were asked to repeat any of those words they could remember both immediately and again after some time. The total score for both word recall tests ranged from 0 to 10. The overall cognitive performance score was calculated as the mean from the standardised score on each test.

Depression was assessed based on 16 questions in the EURO-D instrument. These questions concerned the presence of depressive symptoms, such as pessimism, suicidality, guilt, sleep problems, loss of interest, irritability, changes in appetite, fatigue, and tearfulness in the last month. The EURO-D score ranged from 0 (‘not depressed’) to 12 (‘very depressed’). Respondents were identified as ‘with depression disorder’ if their score was 4 or higher.

Limitation in activities of daily living (ADL) was measured as the number of limitations in any of the ADLs, such as bathing, eating, getting in and out of bed, using the toilet, dressing, or walking across a room. Scores ranged from 0 to 6, a higher score indicating more limitations. The grip strength of both hands was measured twice using a handheld dynamometer (Smedley, S Dynamometer, TTM, Tokyo, 100 kg) (Mehrbrodt et al., 2019). Two measures for one hand were valid when their values differed less than or were equal to 20 kg. In the analysis we used the maximum valid values from both hands' grip strength measurements. This value ranged from 0 to 100 kg. Moderate activity included any activities that required a moderate level of energy such as gardening, cleaning the car, or taking a walk. The frequency of these activities was recorded as ‘>1/week’, ‘1/week’, ‘1–3/month’, and ‘hardly ever/never’.

### 2.4 Statistical analyses

#### 2.4.1 Descriptive analysis

Respondents’ baseline characteristics were described as frequency with percentage or mean with standard deviation. The t-test was used to assess the mean difference in QoL score between baseline and follow-up in the whole sample and by country. The one-way ANOVA test and the t-test were used to assess the mean difference in follow-up CASP score across the different baseline characteristics.

#### 2.4.2 Multivariable linear regression analysis

Multivariable linear regression analysis was used to 1) evaluate the association between participation in social activity, providing, and receiving social support in Wave 6 with QoL (CASP score) in Wave 7 with the other covariates controlled for, and 2) assess non-ignorable dropout. In Model 1 of the multivariable regression, the outcome (QoL in Wave 7) was regressed on the baseline CASP score and the three main exposures of interest (participation in social activity, providing support, and receiving support). The rest of the social relationships and sociodemographic variables were added in Model 2. In Model 3, health-related variables were included. To achieve a more parsimonious model, two insignificant variables (household size and contact with social network members) were dropped from Model 3 (see Equation 1).

Equation 1 $$QoL\;=\;\beta_{0}+\beta_{1}Baseline\;QoL+\beta_{2}Providing\;support+\beta_{3}Receiving\;support+\beta_{4}Social\;participation+\beta_{5}Age+\beta_{6}Men+\beta_{7}Middle\;education\;level+\beta_{8}High\;education\;level+\beta_{9}Retired+\beta_{10}Unable\;to\;work+\beta_{11}Not\;employed+\beta_{12}Make\;ends\;meet\;easily+\beta_{13}Northern+\beta_{14}Central+\beta_{15}Eastern+\beta_{16}Without\;partner+\beta_{17}Number\;of\;children+\beta_{18}Social\;network\;size+\beta_{19}Moderate\;physical\;activity\;1/\;week+\beta_{20}Moderate\;physical\;activity\;1-3\;per\;month+\beta_{21}Moderate\;physical\;activity\;hardly\;ever\;or\;never+\beta_{22}Number\;of\;ADL\;limitations+\beta_{23}Grip\;strength+\beta_{24}Cognitive\;function+\beta_{25}Depressive\;symptom+\varepsilon$$

We also tested interactions between the three main exposures of interest and age, gender, and region, between region and other health and sociodemographic variables, and between perceived household economic status and other sociodemographic variables. We found several significant interaction terms. However, interaction terms between covariates were not included in the final model (Model 3) as they did not meaningfully improve model fit (Tables B1 and B2 in the appendix).

Our analyses did not include 23% (n = 11,306) of respondents who had baseline data but did not have data on QoL at follow-up. This missing data is the result of wave non-response (e.g., respondents did not participate) or item non-response (respondent participated but did not provide a response). In statistical analysis, for convenience, missing data is often ignored under *missing completely at random* (missingness is unrelated to both observed and unobserved data) or *missing at random* (missingness is associated with the observed data but not with the unobserved data) assumptions. However, the more realistic assumption is *missing not at random/non-ignorable* dropout; i.e. data attrition is related to the unobserved data (Feng, Cong, & Silverstein, 2012, pp. 97-136). We therefore present results under this realistic setting using the sensitivity analysis introduced by Genbäck, Stanghellini, & de Luna, 2015.

The dropout is ignorable if the error terms of the outcome regression model and the error terms of the regression model explaining the dropout are unrelated (correlation ρ = 0). The conservative confidence interval is derived under this assumption. The sensitivity analysis yields uncertainty intervals by modelling the dropout as non-ignorable; that is, when the error terms of the two models are correlated. The correlation ρ is allowed to vary within an interval containing zero. We only considered negative correlations (ρ∈[−0.1,0]) because we believe that those who dropped out had a declined QoL (Genbäck, Ng, Stanghellini, & de Luna, 2018).

#### 2.4.3 Doubly robust analyses

We used the counterfactual or potential outcome framework to define causal effects (Holland, 1986) of three exposures of interest (participation in social activity, receiving support, and providing support), separately, on QoL at follow-up. For example, in analysing the first exposure, participation in social activity, we defined two potential outcomes (QoL) for each individual: Y(1), the individual's QoL we would observe if that individual participated in social activity; and Y(0), the QoL we would observe if that same individual did not participate in social activity. The population average causal effect (ACE) of participating in social activity is then defined as E(Y(1)–Y(0)). Similarly, ACEs are defined independently for the other two exposures, receiving and providing support.

**Fig. 1:**
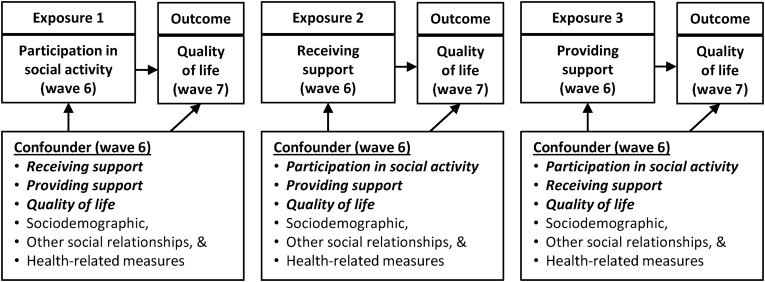
Exposures, outcome, and confounders for each exposure assessment for the whole study population.

The three ACEs defined above were estimated using doubly robust estimators. These separate analyses were performed on the whole study population, as well as sub-populations stratified by age group, gender, and region. A doubly robust estimator combines fitted values from the regression model explaining the outcome for each exposure level with probit regression explaining exposure (propensity score), as described in the appendix (Section C). This approach yields unbiased estimates even when only one of these models is correctly specified (Funk et al., 2011). Only variables in Model 3 were included in the doubly robust estimation. As the three exposures of interest may affect one another, when one exposure was analysed the other two were regarded as confounders (Fig. 1).

To draw causal inferences from observational data, several assumptions are made, including the ignorable exposure assignment (i.e. no unobserved confounders). We performed a sensitivity analysis for the ignorable exposure assignment assumption in a manner similar to that in the sensitivity analysis for the ignorable dropout assumption, according to the approach developed by Genbäck and de Luna (2019). A correlation coefficient (ρ) modelled the non-ignorability of the exposure assignment. Uncertainty intervals with the desired coverage were then obtained by assuming ρ = ∈[−0.05, 0.05], thereby allowing for a small amount of unobserved confounding (Genbäck & de Luna, 2019). Descriptive analyses and multivariable linear regression analyses were conducted using Stata 16.1 (StataCorp, College Station, TX). Doubly robust and both sensitivity analyses were performed using R software version 3.6.1 (R Foundation for Statistical Computing, Vienna; Package ui available on CRAN, https://cran.r-project.org/package=ui).

## 3 Results

### 3.1 Baseline characteristics of study participants

Table 1 shows that women comprised more than half of the sample (56.4%), with an overall mean age of 67.4 years. Approximately a third of the older adults provided support for people from other households, and 21.3% received help from people outside their households. About 56% of respondents did not participate in any of the social activities, 26.9% of respondents participated in one type of activity, and only about 1% participated in all activities. Among those who did participate, the most common activity reported was ‘sport or social club’ (29.8%), followed by ‘voluntary’, ‘educational’, and ‘political’ (7.4%) activity.

**Table 1:** Baseline characteristics of the respondents and mean follow-up CASP-12 score by baseline characteristics (N = 37,908).

	**Baseline characteristics** **(mean±SD or n(%))**	**Follow-up CASP score (mean±SD)**		**Baseline characteristics** **(mean±SD or n(%))**	**Follow-up CASP score (mean±SD)**
***Sociodemographic factors***			***Other social relationship measures***		
Age group***	67.4 ± 9		Marital status***		
50–64	15,615 (41.2)	38.4 ± 5.9	With partner	27,351 (72.2)	37.9 ± 6.1
65–74	13,567 (35.8)	37.9 ± 6.2	Without partner	10,557 (27.9)	36.6 ± 6.5
75+	8,726 (23.0)	35.5 ± 6.6	Number of children**	2.12 ± 1.3	
Gender***			0–2	26,573 (70.1)	37.5 ± 6.2
Woman	21,397 (56.4)	37.2 ± 6.4	≥3	11,335 (29.9)	37.7 ± 6.3
Man	16,511(43.6)	38.0 ± 6.1	Household size***	2.16 ± 1.0	
Education level***			1–2	29,566 (78.0)	37.7 ± 6.3
Low	14,084 (37.2)	35.6 ± 6.6	≥3	8,342 (22.0)	37.2 ± 6.2
Middle	14,810 (39.1)	38.2 ± 5.9	Social network size***	2.72 ± 1.6	
High	9,014 (23.8)	39.5 ± 5.4	0–2	19,100 (50.4)	36.8 ± 6.5
Employment status***			≥3	18,808 (49.6)	38.3 ± 6.0
Employed	9,294 (24.5)	39.5 ± 5.3	Contact with social network members**		
Retired	23,181 (61.2)	37.3 ± 6.2	Never	799 (2.1)	35.0 ± 6.6
Unable to work	844 (2.2)	33.4 ± 6.7	Occasionally	2,330 (6.2)	37.9 ± 6.0
Not employed	4,589 (12.1)	35.6 ± 6.7	Frequently	34,779 (91.8)	37.6 ± 6.3
Household makes ends meet***			***Health-related measures***		
With difficulty	13,293 (35.1)	34.5 ± 6.3	Moderate-level physical activities***		
Easily	24,615 (64.9)	39.2 ± 5.6	>1/week	27,615,(72.9)	38.4 ± 5.9
Region***			1/week	5,181,(13.7)	36.5 ± 6.2
Northern	5,047 (13.3)	40.3 ± 4.9	1-3/month	2,017 (5.3)	35.3 ± 6.4
Central	13,674 (36.1)	39.3 ± 5.6	Hardly ever/never	3,095 (8.2)	32.9 ± 6.9
Eastern	11,026 (29.1)	36.2 ± 6.2	ADL limitationsscore***	0.14 ± 0.6	
Southern	8,161 (21.5)	34.7 ± 6.5	0	34,792 (91.8)	37.9 ± 6.1
***Social support***			≥1	3,116 (8.2)	33.2 ± 6.7
Providing***	11,667(30.8)	38.8 ± 5.6	Grip strength score***	33.7 ± 11.5	
Receiving***	8,086 (21.3)	36.2 ± 6.5	Below mean	19,841 (52.3)	36.6 ± 6.5
***Socially productive activities***			Above mean	18,067 (47.7)	38.6 ± 5.8
Voluntary***	6,665 (17.6)	39.9 ± 5.2	Cognitive function score***	0.11 ± 0.8	
Educational***	4,781 (12.6)	40.2 ± 4.9	Below mean	17,989(47.5)	35.8 ± 6.5
Sport or social club***	11,292 (29.8)	39.9 ± 5.1	Above mean	19,919 (52.6)	39.1 ± 5.5
Political***	2,809 (7.4)	39.5 ± 5.3	Depressive symptomsscore***	2.3 ± 2.1	
At least 1 social activity***	16,707 (44.1)	39.5 ± 5.3	Without depression disorder	28,777 (75.9)	38.8 ± 5.7
			With depression disorder	9,131 (24.1)	33.7 ± 6.5

Note: mean difference of CASP score across baseline characteristics was tested using one-way ANOVA test or t-test. * p value <0.05, ** p value <0.01, *** p value <0.001.

**Fig. 2:**
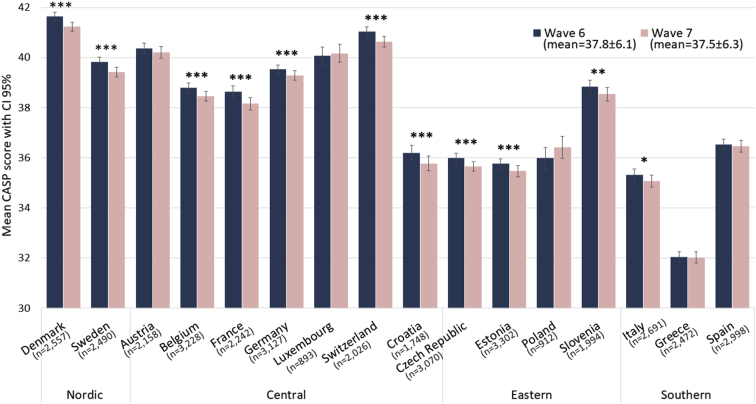
Quality of life across SHARE countries (mean scores of CASP-12 [range 12–48] and confidence intervals 95%) in Wave 6 and Wave 7. Note: * p value <0.05, ** p value <0.01, *** p value <0.001 from paired t-test.

Fig. 2 depicts the levels of QoL score in Waves 6 and 7 by country. Scandinavian countries had a relatively higher QoL score, while Southern European countries had a lower score. The paired t-tests revealed that most of the countries, but not Spain, Greece, Luxembourg, or Poland, had a lower mean QoL score at follow-up.

At follow-up, higher mean CASP scores were observed among those in younger old age, living in Scandinavian countries, with a partner, having high education, employed, with a larger social network size, in contact with their social network members, providing support for other households, participating in socially productive activity, with good physical functioning, and with above average cognitive function scores (Table 1).

### 3.2 Multivariable linear regression results

Model 1 shows that the baseline QoL score together with the three exposures of interest explained around 48% of the variance in the follow-up QoL score, one per cent higher than in the model including only baseline QoL. The size of the association between QoL and the main exposures attenuated after socioeconomic, sociodemographic, and other social relationship variables (Model 2), and health-related variables (Model 3) were added into the models. Compared to the other models, Model 3 had a better fit to the data (higher Adj.R^2^ of 51% and smaller BIC and AIC); see Table 2.

**Table 2:** Multivariable linear regression between quality of life and social participation, providing social support, and receiving social support.

	**Model 1**		**Model 2**		**Model 3**		
	**N = 37,908**		**N = 37,908**		**N = 37,908**		
	**Coeffi-cient**	**95% Confidence Interval**	**Coeffi-cient**	**95% Confidence Interval**	**Coeffi-cient**	**95% Confidence Interval**	**95% Uncertainty Interval**
**Baseline CASP score**	0.673***	(0.665, 0.681)	0.607***	(0.598, 0.615)	0.546***	(0.537, 0.556)	(0.535, 0.556)
**Providing support**	0.580***	(0.479, 0.682)	0.182***	(0.079, 0.285)	0.116*	(0.013, 0.219)	(0.000, 0.219)
**Receiving support**	-0.671***	(-0.783, -0.558)	-0.560***	(-0.674, -0.446)	-0.402***	(-0.516, -0.288)	(-0.516, -0.278)
**Social participation**	1.088***	(0.991, 1.185)	0.587***	(0.486, 0.688)	0.489***	(0.387, 0.590)	(0.364, 0.590)
**Age**			-0.056***	(-0.063, -0.050)	-0.039***	(-0.046, -0.031)	(-0.046, -0.031)
**Man**			0.146**	(0.051, 0.241)	-0.300***	(-0.444, -0.155)	(-0.444, -0.123)
**Education level**							
Middle			0.376***	(0.265, 0.486)	0.279***	(0.168, 0.389)	(0.168, 0.396)
High			0.501***	(0.371, 0.630)	0.361***	(0.229, 0.493)	(0.229, 0.501)
**Employment status**							
Retired			0.019	(-0.116, 0.155)	0.007	(-0.127, 0.142)	(-0.151, 0.142)
Unable to work			-1.908***	(-2.224, -1.593)	-1.353***	(-1.670, -1.037)	(-1.681, -1.037)
Not employed			-0.286**	(-0.458, -0.114)	-0.234**	(-0.404, -0.063)	(-0.414, -0.063)
**Household makes ends meet easily**			0.768***	(0.659, 0.877)	0.742***	(0.633, 0.850)	(0.629, 0.850)
**Region**							
Northern			1.091***	(0.912, 1.269)	1.092***	(0.912, 1.271)	(0.912, 1.275)
Central			0.952***	(0.812, 1.092)	1.061***	(0.919, 1.203)	(0.912, 1.203)
Eastern			0.338***	(0.202, 0.474)	0.306***	(0.167, 0.445)	(0.134, 0.445)
**Without partner**			-0.226***	(-0.339, -0.113)	-0.219***	(-0.323, -0.115)	(-0.328, -0.115)
**Number of children**			0.045*	(0.009, 0.080)	0.044*	(0.010, 0.079)	(0.004, 0.079)
**Household size**			-0.013	(-0.067, 0.041)			
**Social network size**			0.098***	(0.067, 0.129)	0.105***	(0.075, 0.135)	(0.068, 0.135)
**Contact with social network members**							
Occasionally			0.218	(-0.151, 0.587)			
Frequently			0.367*	(0.046, 0.689)			
**Moderate-level physical activities**							
1/week					-0.294***	(-0.426, -0.162)	(-0.427, -0.162)
1-3/month					-0.425***	(-0.626, -0.225)	(-0.626, -0.204)
Hardly ever/never					-0.514***	(-0.692, -0.336)	(-0.692, -0.315)
**ADL limitationsscore**					-0.403***	(-0.487, -0.32)	(-0.487, -0.308)
**Grip strength**					0.019***	(0.013, 0.026)	(0.012, 0.026)
**Cognitive function score**					0.181***	(0.111, 0.251)	(0.089, 0.251)
**Depressive symptom score**					-0.225***	(-0.250, -0.199)	(-0.250, -0.198)
**Constant**	11.58***	(11.29, 11.87)	16.25***	(15.61, 16.90)	17.88***	(17.22, 18.54)	(17.22, 18.87)
**Adjusted R-squared**	0.484		0.502		0.511		
**BIC**	221488		220292		219681		
**AIC**	221445		220104		219459		

Note: * p value <0.05, ** p value <0.01, *** p value <0.001. CASP=Control Autonomy Self-realisation Pleasure (measure of QoL), ADL = Activities of Daily Living, BIC=Bayesian Information Criterion, AIC = Akaike Information Criterion. Reference categories for categorical variables: education level (low), employment status (employed), household economic situation (with difficulty), regions (southern), marital status (with partner), average frequency of contact with social network members (never), moderate-level physical activities (>1/week).

Results from Model 3 show that the baseline QoL score had a positive association with the follow-up QoL (0.55, 95%CI:0.54,0.56). Participation in social activities and providing social support were also positively associated with QoL at follow-up (0.49, 95%CI:0.39,0.59 and 0.12, 95%CI:0.013,0.22, respectively). In contrast, receiving support at baseline was negatively associated with QoL at follow-up (-0.40, 95%CI:-0.52,-0.29).

The uncertainty intervals taking into account our uncertainty regarding non-ignorable dropout for Model 3 are presented in Table 2. The 95% CI and 95% UI for each estimate in this model lead to the same conclusion. Thus, we can conclude that these estimates were not sensitive to the amount of non-ignorable dropout considered.

### 3.3 Doubly robust analysis results

The estimates of ACE and their 95% CI and UI are presented in Table 3. The ACE of providing support was not significant (ACE:0.112,95%CI:-0.03,0.253). Participation in social activities and QoL had a significant positive association (ACE:0.474, 95%CI:0.361,0.587), while receiving support was negatively associated with QoL (ACE:-0.321, 95%CI:-0.448,-0.195). Note that the UI for each of these associations includes zero, meaning that if there are unobserved confounders, we cannot infer any causal interpretation of the associations observed.

**Table 3:** Average causal effect (ACE) of receiving social support, providing social support, and social participation on quality of life, on average, by region, gender, and age group (N = 37,908).

	**Social participation**			**Receiving support**			**Providing support**		
	**ACE**	**95% Confidence Interval**	**95% Uncertainty Interval**	**ACE**	**95% Confidence Interval**	**95% Uncertainty Interval**	**ACE**	**95% Confidence Interval**	**95% Uncertainty Interval**
**Whole study population**	**0.474**	**(0.361, 0.587)**	(-0.001, 0.949)	**-0.321**	**(-0.448, -0.195)**	(-0.840, 0.197)	0.112	(-0.03, 0.253)	(-0.386, 0.609)
**Region**									
Northern	**0.219**	**(0.008,0.429)**	(-0.294, 0.731)	-0.133	(-0.365, 0.099)	(-0.664, 0.397)	0.195	(-0.007, 0.397)	(-0.274, 0.664)
Central	**0.456**	**(0.313,0.599)**	(-0.012, 0.924)	-0.166	(-0.348, 0.016)	(-0.705, 0.373)	0.135	(-0.023, 0.293)	(-0.337, 0.607)
Eastern	**0.570**	**(0.328,0.813)**	(-0.059, 1.200)	**-0.737**	**(-0.960,-0.513)**	**(-1.364,-0.109)**	-0.188	(-0.507, 0.131)	(-0.903, 0.527)
Southern	**0.657**	**(0.302,1.012)**	(-0.142, 1.456)	-0.055	(-0.487, 0.377)	(-0.987, 0.876)	0.153	(-0.255, 0.560)	(-0.714, 1.019)
**Gender**									
Man	**0.386**	**(0.220,0.551)**	(-0.140, 0.911)	**-0.437**	**(-0.643,-0.231)**	(-1.038, 0.164)	0.087	(-0.123, 0.297)	(-0.478, 0.652)
Woman	**0.546**	**(0.395,0.698)**	**(0.030,1.063)**	**-0.243**	**(-0.404,-0.081)**	(-0.792, 0.307)	0.139	(-0.057, 0.335)	(-0.415, 0.693)
**Age group**									
50–64	**0.420**	**(0.258,0.582)**	(-0.101, 0.941)	**-0.226**	**(-0.433,-0.018)**	(-0.821, 0.370)	0.108	(-0.051, 0.268)	(-0.394, 0.610)
65–74	**0.487**	**(0.302,0.672)**	(-0.056, 1.030)	**-0.256**	**(-0.473,-0.039)**	(-0.858, 0.347)	-0.019	(-0.219, 0.181)	(-0.570, 0.532)
75+	**0.546**	**(0.263,0.829)**	(-0.122, 1.213)	**-0.548**	**(-0.778,-0.318)**	(-1.171, 0.075)	**0.410**	**(0.031,0.789)**	(-0.367, 1.187)

Note: 95% CI and 95% UI that do not include zero (significant) and their corresponding ACE estimates are bolded.

The results from sub-population analysis by region, gender, and age group (Table 3) revealed that providing support had a significant positive association with QoL only among respondents aged 75+. On the other hand, receiving social support had a significant negative association with QoL in the Eastern European region and in all sub-groups by gender and age group. This association was stronger among men compared to women, and among the older age group.

Participation in social activities was positively associated with QoL in all sub-populations. We observed a gradient of the strength of association (i.e. size of the ACE estimates) of social participation across regions. The weakest association was observed in Northern European countries and the strongest in Southern European countries. The association was stronger among women and people aged 75+.

In general, the strength of associations between the three exposures and QoL was moderate and significant. The size of ACE estimates of receiving social support ranged from -0.23 to -0.74, while the corresponding estimates of social participation ranged from 0.22 to 0.66. Stratification of the analyses by other sociodemographic factors, e.g., education level, yielded in ACE estimates within the aforementioned ranges (Table C1 in the appendix). Most of the significant ACE estimates from the sub-population analyses had UI that included zero, only receiving social support in Eastern European countries and social participation among women did not. Thus, in general, no causal interpretation is justified by our sensitivity analysis due to potential unobserved confounders.

## 4 Discussion

A vast body of literature provides evidence that social participation and social support are positively associated with older adults’ health and subjective QoL (e.g. Inagaki and Orehek (2017), Gariépy et al. (2016), Wahrendorf and Siegrist (2010)). The mechanisms behind these associations are highly complex (Berkman & Krishna, 2014, pp. 273–319; Liang, Krause, & Bennett, 2001); different conceptualisations and operationalisations of social support, participation, and QoL might lead to a different conclusion. In addition, as longitudinal studies in this field of research are scarce, much of the evidence originates from cross-sectional analyses (Liang, Krause, & Bennett, 2001; Turner & Turner, 2013, pp. 341-356).

The present study sought to contribute to the evidence on how each exposure – i.e. social participation, providing instrumental support, and receiving instrumental support – was associated with (or had a causal effect on) QoL in European older adults. We approached this objective by testing the associations between the three exposures of interest and QoL using multivariable linear regression and by evaluating whether there was evidence of causal effects using doubly robust estimation and a sensitivity analysis for unobserved confounding. We observed a positive association between social participation and QoL and a negative association between receiving instrumental support and QoL. The sensitivity analysis indicated that most of these associations cannot be interpreted as causal. We also showed the heterogeneity in the association between the three exposures and outcome across sub-populations by age group, gender, and region.

QoL in older people varied across countries. SHARE data showed that older people in Southern European nations (e.g. Italy and Spain) consistently reported lower QoL compared to their counterparts in the Scandinavian countries (Litwin & Stoeckel, 2013; Wahrendorf et al., 2006; Wahrendorf & Siegrist, 2010). Similar findings were also reported in the ABUEL (Abuse of the elderly in the European region) study; i.e., older adults in Italy, Portugal, Lithuania, and Greece had lower QoL than older adults in Spain, Germany, and Sweden (Soares et al., 2010). In line with what these previous studies have described, the level of QoL was highest in the Northern European region, and lowest in the Southern European region. The cross-country differences in QoL may reflect the cultural, political, and other contextual factors that varied across the European countries. For instance, each country differs in its capacity and commitment to provide healthcare services, as well as social and economic support (welfare policies) for older adults, thereby possibly affecting QoL in older people (Ioannidi & Mestheneos, 2013, pp. 151-162; Motel-Klingebiel et al., 2009). The 2016 European Quality of Life Survey report indicated this association between QoL and public service quality. Many Southern European countries (e.g. Italy and Greece) ranked relatively low in terms of perceived quality of overall public services, such as healthcare, long-term care, education, social housing, and pension system. The Eastern European countries (e.g. Czech Republic and Estonia) ranked relatively higher, but still lower than countries in Central (e.g. Austria and Luxemburg), and Northern Europe (e.g. Sweden and Denmark) (Eurofound, 2017). A similar pattern was observed regarding the level of income insecurity in old age (Eurofound, 2017). Additionally, we have to consider the possible bias due to culture and language-specific interpretation of the CASP-12 items (Tesch-Römer, Motel-Klingebiel, & von Kondratowitz, 2003, pp. 275-280).

### 4.1 The overall association between QoL and participation in social activities as well as providing and receiving support

Our study indicates that participation in at least one of four types of social activities (voluntary/charity work, educational/training course, sport/social club, and political/community organisation) was positively associated with QoL in older European adults. These findings are in line with previous studies on social participation, e.g. Newman et al. (2014). The positive association between social participation and QoL is supported by the activity theory of ageing. This theory postulates that, by staying active by retaining or replacing their middle-age social activities, older adults can maintain their personal identity, self-esteem, life satisfaction, and well-being (Bengtson, 2016, pp. 67–86; Diggs, 2008, pp.79-81). Moreover, participation in social activity may promote social integration and improve one's social capital and healthy behaviours, which in turn may promote better QoL (Berkman & Krishna, 2014, pp. 273–319).

It has been suggested that social activities lead to positive well-being, particularly when they are perceived as leisure activities (Newman et al., 2014). Leisure activities may influence QoL through several psychological mechanisms, namely detachment (from work)-recovery, autonomy, mastery, meaning, and affiliation (Newman et al., 2014). Engagement in voluntary activity may increase mastery and add meaning to one's life (Newman et al., 2014), distract from one's own problems, and improve one's mood (Post, 2005). Educational leisure activities may additionally benefit QoL by increasing mastery and promoting cognitive function (Simone & Cesena, 2010). Physical leisure activities, such as playing sports, may promote QoL via the detachment-recovery process and by fulfilling the needs for affiliation and mastery (Newman et al., 2014). Playing sports may also maintain physical function (Manini & Pahor, 2009) and delay the onset or progression of ADL limitation (Tak et al., 2013). Moreover, it is important to note that the effect on QoL of volunteering, providing instrumental support, and providing personal care may depend on the extent of autonomy and perceived control in these activities (Wahrendorf et al., 2008).

Our findings also concur with previous research which found a negative association between receiving support and QoL (Liang, Krause, & Bennett, 2001; Litwin & Stoeckel, 2013; Tesch-Römer, Motel-Klingebiel, & von Kondratowitz, 2003, pp. 275-280). On the other hand, we observed a lack of association between providing support and QoL. Two theories, namely the equity theory and the esteem enhancement theory, may explain the effect of social support on health and QoL. The equity theory (Walster, Walster, & Berscheid, 1978) posits that those who receive more support than they can provide violate the norm of reciprocity and may be distressed or feel guilty. Also, people who give more than they receive in return may experience negative emotions. Both these conditions may negatively affect QoL. The esteem enhancement theory (DuBois, Flay, & Fagen, 2009, pp. 98–130) suggests that the effect of social support on health and well-being is moderated by the appraisal of self-esteem/self-worth. Providing help to someone in need may lead to a positive appraisal of one's self-worth, in turn increasing QoL. Recipients of support may interpret the received support as evidence of care and love, thus enhancing their self-esteem (Dykstra, 2015, pp. 88-93); meanwhile, for others, receiving support may damage their self-esteem if they perceive this as evidence of their failure and inability to function independently (loss of autonomy), thereby lowering their self-esteem and resulting in lower QoL (Dykstra, 2015, pp. 88-93; Tesch-Römer, Motel-Klingebiel, & von Kondratowitz, 2003, pp. 275-280).

While we are able to rule out possible reverse causation as the explanation for this association, we acknowledge the possible influences of unmeasured confounders. Hence, we assumed that the negative association between receiving support and QoL was partly due to receiving support capturing some effects of poor health that were not measured by our health-related variables (grip strength, ADL limitations, and depression symptoms) at baseline (Seidman et al., 2006; Tesch-Römer, Motel-Klingebiel, & von Kondratowitz, 2003, pp. 275-280). It is important to note that this negative association did not imply the effectiveness of the help received; it may actually be helpful to the recipient and they may feel grateful for it, while at the same time feeling that accepting help hurts their sense of control and autonomy.

One possible explanation for the weak association between providing support and QoL is that SHARE assessed the provision of several types of support in one measure (e.g., personal care, practical household help, and help with paperwork), each of which may have a different association with QoL. As shown in previous research, providing personal care tends to be associated with poorer QoL, whereas other informal help has a positive association (Wahrendorf et al., 2006). Furthermore, as shown by our multivariable linear regression analyses, health-related variables confound the association observed between the exposures of interest and QoL. The confounding effect seems to be stronger on the association between providing support and QoL. This indicates that those among our study population who provided help to others were mostly healthy; and health status was a strong determinant of QoL at two-year follow-up.

### 4.2 The heterogeneity of the association between QoL and participation in social activities as well as providing and receiving support, by gender, age group, and region

As expected, even though the directions of association between each of our three exposures and QoL were identical, the strength varied across sub-populations. We observed that the ACE estimates of social participation were higher in sub-populations with lower participation levels (Table D1 in the appendix). There are two possible explanations for this. First, in some societies, participation in sport, education, and voluntary activities is likely to be associated with individualism and an economically privileged status, which can lead to better QoL. Thus, larger ACE estimates that were found among older people in countries where social participation was less prevalent likely because those who were able to participate were more privileged in a way that was not measured in this study. Second, the relative standards theory suggests that the effect of objective conditions on well-being is determined through comparison with other possible conditions (Diener & Lucas, 2000). Thus, people who were able to participate in social activities judge their QoL better after comparing themselves with other people in their population who were not able to participate. This could also explain the larger ACE estimates but lower participation among women and the older age group observed in this study.

The significant association between providing support and QoL in the oldest age group (75+) was quite unexpected, considering the strong confounding effect of health factors on providing support demonstrated in the whole study population analysis. Also, health problems are expected to be more common in the older group. The esteem enhancement theory may help explain this positive association; that is, providing support to others in need may increase self-esteem, which in turn results in higher QoL. It is possible that the ‘esteem enhancement’ was more significant in the oldest age group, as this group is often characterised by poor health and lower economic status (conditions that can lower self-esteem). Along the same lines, an explanation based on the relative standards theory is also possible (Diener & Lucas, 2000); that is, older adults in this group assessed their QoL more positively after comparing themselves with their counterparts who were less likely to be able to provide support.

The stronger negative association of receiving support among men was anticipated. The cultural constructions of femininities and masculinities may lead men to appraise the support they receive more negatively than women. Furthermore, as suggested by the esteem enhancement theory, negative appraisal of receiving support may result in lower self-esteem that in turn leads to lower QoL. This theory may also explain the stronger negative association of receiving support in the older age group. In this case, the negative evaluation of receiving support may be more harmful to self-esteem among this more vulnerable sub-population. However, it is also possible that the effect of unmeasured health-related confounders was more prominent in the older group. Furthermore, drawing from the equity theory, the fact that those in the older age group tend to require more, but provide less, support may make them feel as if they are violating the norm of reciprocity, leading to negative emotion that in turn may result in lower QoL (Gleason et al., 2008; Liang, Krause, & Bennett, 2001).

Interestingly, we only observed a significant negative association between receiving support and QoL in the Eastern region. This finding may reflect the influence of contextual factors such as cultural norms, family values, and care service availability that shape the typology of social support exchange across Europe. In Southern and Eastern European countries, family norms such as filial obligation are stronger and care service is limited. On the other hand, family obligation is less demanding in the more developed welfare state, such in Northern and Central Europe (Daatland et al., 2011). Therefore, it is possible that in a society that demands support from family, older adults negatively value receiving support from people outside their household. Also, we cannot exclude the possible effect of unobserved health-related confounding factors in this sub-population. Nonetheless, it remains unclear why, in the other region with similar family values (i.e., the Southern region), no association was found between receiving support and QoL. Studies are needed in order to explore which contextual factors determine the association between social participation, social support, and QoL, and how.

### 4.3 Causal interpretation

In this study, even though we established the time sequence of the exposure and outcome, controlled for various potential confounders, and implemented a robust statistical method (Funk et al., 2011), we cannot ignore the possible unobserved residual confounders. The assumption of no unobserved confounders (random assignment of exposure) is essential in drawing causal inference. However, as it is untestable empirically, we performed a sensitivity analysis producing uncertainty intervals (UIs) to help us evaluate whether the associations obtained are sensitive to unobserved confounders (Genbäck & de Luna, 2019). The UIs included zero for almost all estimators presented in Table 3. Therefore, we cannot make a causal interpretation of the estimated associations. There are several factors that may have confounded the association between our exposures and QoL, such as personality traits (Siedlecki et al., 2014; Steel, Schmidt, & Shultz, 2008), motivation behind social participation or support exchange (Dykstra, 2015, pp. 88-93), and expectation of support (Liang, Krause, & Bennett, 2001), which are yet to be explored in future studies.

Finding empirical evidence for causal effects of social relationships on QoL is indeed challenging. This is due to the complexities of possible pathways behind this association, and difficulties in establishing the proper follow-up time between the exposures and outcome (Liang, Krause, & Bennett, 2001). In our case, the two-year interval between waves may have been too long to ascertain the effect of social support and participation assessed at baseline on QoL at follow-up. A shorter follow-up may have allowed us to observe stronger associations and to rule out the potential influence of other variables. During a long follow-up time some changes in key factors may occur and act as moderators or mediators between the exposure and outcome of interest, making it difficult to ascertain the exposure's main effect.

### 4.4 Strengths and limitations

Besides the limitations related to the study design and method that we discussed above; another limitation involves the measurement of some variables in this study. The CASP scale is based on theoretical constructs rather than a subject's account, and thus may miss some QoL domains (Hyde, Wiggins, Higgs, & Blane, 2003). The types of social activities assessed in this study may not represent the common activities in all the countries studied. Additionally, SHARE measures each of the main exposures used in this study using a single-item measure; it is possible that a more comprehensive measure would produce different results. In addition, incorporating indicators of the quality (e.g. frequency, duration, satisfaction) of social activities and social support exchange in the analysis may improve the QoL prediction model (Siegrist & Wahrendorf, 2009).

One of the strengths of this study comes from its use of SHARE data. In all SHARE participating countries, a vigorously controlled study protocol was implemented, study instruments were translated into different languages following standard procedure, data collection was performed by trained interviewers, and data was carefully checked. The SHARE response rate was quite high, but varied across the participating countries (Börsch-Supan et al., 2013). Thus, we cannot rule out the possible effect of selection bias on the results of this study. Another strength of this study is the use of doubly robust estimation and sensitivity analysis to evaluate whether we can make causal interpretations based on the associations observed between the exposures and QoL.

Despite its limitations, this study makes several unique contributions. By addressing the temporality issue between exposure and outcome, using doubly robust estimators, and evaluating the results’ sensitivity to non-ignorable dropout and unobserved confounders, we offer stronger evidence that social participation and receiving support are, independently, associated with QoL in European older adults at two-year follow-up. These associations, however, cannot be interpreted as causal effects.

## 5 Conclusions

This study found a positive association between social participation and QoL, and a negative association between receiving instrumental support and QoL. The strength of these associations varies across sub-populations by age group, gender, and region. The positive association between providing support and QoL was only significant in the oldest age group. Despite the associations found, the sensitivity analysis indicated that most of these associations should not be interpreted as causal effects.

Considering that social participation and social support are important parts of healthy ageing policy in Europe, more research is needed to clarify the underlying mechanisms linking social participation, social support, and older adults’ QoL. Data from a well-designed population-based longitudinal study is needed to determine the direction of causation between social participation, social support, and QoL. Furthermore, future research needs to evaluate and recommend evidence-based policies and interventions for improving QoL in older populations.

Our results also showed the importance of sub-population analysis, especially when the goal is to develop an intervention programme. Further investigations are needed to evaluate how different types of social activity as well as receiving and providing support influence QoL in older adults of different ages and genders, and in different settings. Such studies may explain the mechanisms behind the negative and positive associations between receiving or providing support and QoL.

Meanwhile, the lack of causal interpretation of the effect of social participation and social support on QoL in this study should not discourage older adults from participating in social activities, providing support, or receiving support. Instead, the observed negative association between receiving support and QoL should encourage care providers to deliver support in ways that respect recipients’ autonomy and control.

## Declaration of competing interest

The authors declare that they have no known competing financial interests or personal relationships that could have appeared to influence the work reported in this paper.

## Data Availability

SHARE data is freely available for scientific community after registration. This data is distributed by SHARE-ERIC (Survey of Health, Ageing and Retirement in Europe – European Research Infrastructure Consortium). All data user is subject to European Union and national data protection laws and the SHARE Conditions of Use. More details on data access is available in http://www.share-project.org/data-access.html.
