# Supplementary material for: A longitudinal study on social support, social participation, and older Europeans’ Quality of life
Source: SSM Popul Health. 2021 Feb 3;13:100747. doi: 10.1016/j.ssmph.2021.100747 (PMC7892994; doi:10.1016/j.ssmph.2021.100747)
Supplement: Multimedia component 1 [file mmc1.pdf]

Appendix

A. CASP-12 Questionnaire

Table A1. CASP-12 Questionnaire

Question text	Domain
How often do you think your age prevents you from doing the things you would like to do?	Control
How often do you feel that what happens to you is out of your control?	Control
How often do you feel left out of things?	Control
How often do you think that you can do the things that you want to do?	Autonomy
How often do you think that family responsibilities prevent you from doing what you want to do?	Autonomy
How often do you think that shortage of money stops you from doing the things you want to do?	Autonomy
How often do you look forward to each day?	Pleasure
How often do you feel that your life has meaning?	Pleasure
How often, on balance, do you look back on your life with a sense of happiness?	Pleasure
How often do you feel full of energy these days?	Self-Realization
How often do you feel that life is full of opportunities?	Self-Realization
How often do you feel that the future looks good for you?	Self-Realization

Source: https://www.share-datadocutool.org/control_construct_schemes/view/189

B. Additional multivariable linear analyses

Table B1. Multivariable linear regression of quality of life on baseline at Wave 6 with interaction terms (between the main exposures with age, gender, and region; region with other covariates).

	Model 4 (N= 37.908)	
	Coefficient	95%CI
Baseline CASP score	0.426***	(0.406,0.445)
Participation in social activities	0.302	(-0.467,1.070)
Providing support	-0.776	(-1.600,0.048)
Receiving support	0.436	(-0.443,1.316)
Age	-0.078***	(-0.091,-0.065)
Man	0.456**	(0.169,0.744)
Education level		
Middle	-0.166	(-0.413,0.081)
High	0.156	(-0.145,0.457)
Employment status		
Retired	-0.066	(-0.203,0.071)
Unable to work	-0.249**	(-0.422,-0.075)
Not employed	-1.316***	(-1.633,-1.000)
Household make ends meet easily	1.560***	(1.348,1.771)
Region		
Northern	-7.639***	(-10.067,-5.212)
Central	-9.686***	(-11.362,-8.011)
Eastern	-4.687***	(-6.418,-2.956)
Without Partner	-0.201***	(-0.306,-0.097)
Number of children	0.037*	(0.003,0.072)
Social network size	0.233***	(0.165,0.301)
Moderate level physical activities		
>1/week	-0.294***	(-0.425,-0.163)
1-3/month	-0.411***	(-0.611,-0.211)
Hardly ever/never	-0.533***	(-0.711,-0.356)
Number of ADL limitations	-0.376***	(-0.459,-0.292)
Grip strength	0.003	(-0.010,0.016)
Cognitive function score	0.499***	(0.336,0.661)
Depressive symptom score	-0.181***	(-0.228,-0.133)
**Man x participation in social activity**	-0.139	(-0.333,0.054)
**Man x providing support**	0.059	(-0.143,0.261)
**Man x receiving support**	-0.201	(-0.426,0.025)
**Age x participation in social activity**	0.004	(-0.007,0.015)
**Age x providing support**	0.013*	(0.001,0.025)
**Age x receiving support**	-0.012	(-0.023,0.000)
**Region x participation in social activity**		
Central x yes	-0.038	(-0.322,0.246)
Northern x yes	-0.294	(-0.662,0.074)
Eastern x yes	0.101	(-0.203,0.405)

Table B1. (continued)

	Model 4 (N= 37.908)	
	Coefficient	95%CI
**Region x providing support**		
Central x yes	0.046	(-0.271,0.363)
Northern x yes	0.072	(-0.304,0.447)
Eastern x yes	-0.046	(-0.382,0.289)
**Region x receiving support**		
Central x yes	0.224	(-0.129,0.577)
Northern x yes	0.377	(-0.037,0.792)
Eastern x yes	-0.286	(-0.642,0.071)
**Region x baseline CASP score**		
Central x baseline CASP score	0.188***	(0.162,0.215)
Northern x baseline CASP score	0.210***	(0.174,0.246)
Eastern x baseline CASP score	0.088***	(0.062,0.114)
**Region x gender**		
Central x man	-0.735***	(-1.113,-0.357)
Northern x man	-0.669*	(-1.188,-0.150)
Eastern x man	-1.167***	(-1.551,-0.783)
**Region x age**		
Central x age	0.065***	(0.049,0.082)
Northern x age	0.033**	(0.010,0.055)
Eastern x age	0.028**	(0.011,0.045)
**Region x education**		
Central x middle	0.497**	(0.186,0.808)
Central x high	0.136	(-0.235,0.507)
Northern x middle	0.166	(-0.246,0.578)
Northern x high	-0.025	(-0.479,0.428)
Eastern x middle	0.671***	(0.360,0.982)
Eastern x high	0.581**	(0.184,0.978)
**Region x household makes end meet**		
Central x easily	-0.843***	(-1.134,-0.551)
Northern x easily	-1.319***	(-1.781,-0.857)
Eastern x easily	-1.111***	(-1.384,-0.838)
**Region x social network size**		
Central x social network size	-0.189***	(-0.271,-0.107)
Northern x social network size	-0.159**	(-0.262,-0.056)
Eastern x social network size	-0.106*	(-0.195,-0.017)
**Region x grip strength**		
Northern x grip strength	0.012	(-0.010,0.035)
Central x grip strength	0.016	(-0.001,0.033)
Eastern x grip strength	0.037***	(0.020,0.055)

Table B1. (continued)

	Model 4 (N= 37.908)	
	Coefficient	95%CI
**Region x cognitive function**		
Central x cognitive function	-0.280**	(-0.480,-0.079)
Northern x cognitive function	-0.24	(-0.491,0.012)
Eastern x cognitive function	-0.547***	(-0.751,-0.343)
**Region x depressive symptom**		
Central x with depression disorder	-0.062	(-0.126,0.003)
Northern x with depression disorder	-0.079	(-0.172,0.014)
Eastern x with depression disorder	-0.075*	(-0.141,-0.010)
Constant	24.474***	(23.188,25.759)
Adjusted R-squared	0.518	
BIC	219583	
AIC	218977	

Note: * p value < 0.05, ** p value <0.01, *** p value <0.001. CASP= Control Autonomy Self-realization Pleasure (measure of quality of life), ADL = Activity of Daily Living, BIC = Bayesian Information Criterion, AIC = Akaike Information Criterion. Reference categories for categorical variables: regions(southern), average frequency of contact with social network members (never), education level (low), household economic situation (with difficulty), employment status (employed), moderate level physical activities (>1/ week).

Table B2. Multivariable linear regression of quality of life on baseline at wave six with interaction terms (between perceived household economic status with other sociodemographic covariates).

	Model 5 (N= 37.908)	
	Coefficient	95%CI
Baseline CASP score	0.544***	(0.534,0.554)
Participated in social activities	0.495***	(0.394,0.596)
Providing support	0.119*	(0.016,0.222)
Receiving support	-0.397***	(-0.511,-0.283)
Household make ends meet easily	1.170**	(0.319,2.020)
Age	-0.034***	(-0.045,-0.024)
Man	-0.186	(-0.380,0.007)
Education level		
Middle	0.289**	(0.116,0.462)
High	0.363**	(0.123,0.603)
Without partner	-0.282**	(-0.452,-0.112)
Employment status		
Retired	-0.257*	(-0.497,-0.017)
Not employed	-0.484***	(-0.744,-0.225)
Unable to work	-1.411***	(-1.843,-0.980)
Region		
Central	0.936***	(0.706,1.166)
Northern	1.431***	(1.015,1.847)
Eastern	0.608***	(0.419,0.797)

Table B2. **(continued)**

	Model 5 (N= 37.908)	
	Coefficient	95%CI
**Household make ends meet easily x age**	-0.006	(-0.019,0.007)
**Household make ends meet easily x men**	-0.181	(-0.379,0.016)
**Household make ends meet easily x middle**	-0.01	(-0.231,0.212)
**Household make ends meet easily x high**	0.013	(-0.268,0.295)
**Household make ends meet easily x without partner**	0.114	(-0.098,0.327)
**Household make ends meet easily x retired**	0.371*	(0.081,0.662)
**Household make ends meet easily x not employed**	0.438*	(0.088,0.789)
**Household make ends meet easily x unable to work**	-0.028	(-0.662,0.607)
**Household make ends meet easily x central**	0.051	(-0.233,0.335)
**Household make ends meet easily x northern**	-0.476*	(-0.933,-0.020)
**Household make ends meet easily x eastern**	-0.616***	(-0.885,-0.348)
Number of children	0.046**	(0.011,0.081)
Social network size	0.104***	(0.074,0.134)
Number of ADL limitations	-0.406***	(-0.490,-0.322)
Moderate level physical activities		
1/week	-0.281***	(-0.413,-0.150)
1-3/month	-0.403***	(-0.604,-0.203)
Hardly ever/never	-0.511***	(-0.688,-0.333)
Depressive symptom score	-0.226***	(-0.251,-0.200)
Cognitive function score	0.185***	(0.115,0.255)
Grip strength	0.020***	(0.014,0.027)
Constant	17.728***	(16.886,18.569)
Adjusted R-squared	0.512	
BIC	219746	
AIC	219430	

Note: * p value < 0.05, ** p value <0.01, *** p value <0.001. CASP= Control Autonomy Self-realization Pleasure (measure of quality of life), ADL = Activity of Daily Living, BIC = Bayesian Information Criterion, AIC = Akaike Information Criterion. Reference categories for categorical variables: regions(southern), average frequency of contact with social network members (never), education level (low), household economic situation (with difficulty), employment status (employed), moderate level physical activities (>1/week).

C. Causal effects and doubly robust estimation

We use the potential outcome framework to formalise the causal effect estimated and the models used. Let $Z \in \{0,1\}$ be the exposure / treatment of interest. Thus, Z= 1 in the presence of exposure (e.g. "participation in social activity") and Z= 0 in the absence of exposure ("no participation in social activity"). Then, for each individual i in the study, two potential outcomes (QoL) are defined as follow:

$Y_{(1)}$: QoL at follow-up would the individual participate (Z= 1)

$Y_{(0)}$: QoL at follow-up would the individual not participate (Z= 0)

The causal effect of interest in this study is τ = E ($Y_{(1)} - Y_{(0)}$), i.e. the average causal effect of the exposure on QoL. We then follow Genbäck and de Luna (2019) to model the two potential outcomes with regression models:

$$
\begin{aligned}
Y_{(1)} = \gamma_{10} &+ \gamma_{11} Baseline\ QoL + \gamma_{12} Providing\ sopport + \gamma_{13} Receiving\ support + \gamma_{14} Age + \gamma_{15} Man \\
&+ \gamma_{16} Middle\ education\ level + \gamma_{17} High\ education\ level + \gamma_{18} Retired \\
&+ \gamma_{19} Unable\ to\ work + \gamma_{110} Not\ employed + \gamma_{111} Make\ ends\ meet\ easily + \gamma_{112} Northern \\
&+ \gamma_{113} Central + \gamma_{114} Eastern + \gamma_{115} Without\ partner + \gamma_{116} Number\ of\ children \\
&+ \gamma_{117} Social\ network\ size + \gamma_{118} Moderate\ physical\ activity\ 1/week \\
&+ \gamma_{119} Moderate\ physical\ activity\ 1-3\ per\ month \\
&+ \gamma_{120} Moderate\ physical\ activity\ hardly\ ever\ or\ never \\
&+ \gamma_{121} Number\ of\ ADL\ limitations + \gamma_{122} Grip\ strength + \gamma_{123} Cognitive\ function \\
&+ \gamma_{124} Depressive\ symptom + \varepsilon_{(1)}
\end{aligned}
$$

Equation C1

$$
\begin{aligned}
Y_{(0)} = \gamma_{00} &+ \gamma_{01} Baseline\ QoL + \gamma_{02} Providing\ sopport + \gamma_{03} Receiving\ support + \gamma_{04} Age + \gamma_{05} Man \\
&+ \gamma_{06} Middle\ education\ level + \gamma_{07} High\ education\ level + \gamma_{08} Retired \\
&+ \gamma_{09} Unable\ to\ work + \gamma_{010} Not\ employed + \gamma_{011} Make\ ends\ meet\ easily + \gamma_{012} Northern \\
&+ \gamma_{013} Central + \gamma_{014} Eastern + \gamma_{015} Without\ partner + \gamma_{016} Number\ of\ children \\
&+ \gamma_{017} Social\ network\ size + \gamma_{018} Moderate\ physical\ activity\ 1/week \\
&+ \gamma_{019} Moderate\ physical\ activity\ 1-3\ per\ month \\
&+ \gamma_{020} Moderate\ physical\ activity\ hardly\ ever\ or\ never \\
&+ \gamma_{021} Number\ of\ ADL\ limitations + \gamma_{022} Grip\ strength + \gamma_{023} Cognitive\ function \\
&+ \gamma_{024} Depressive\ symptom + \varepsilon_{(0)}
\end{aligned}
$$

Equation C2

We also need to model the exposure given baseline covariates using a probit regression model:

$$\begin{aligned} Z^* = {} & \delta_0 + \delta_1 Baseline\ QoL + \delta_2 Providing\ sopport + \delta_3 Receiving\ support + \delta_4 Age + \delta_5 Man \\ & + \delta_6 Middle\ education\ level + \delta_7 High\ education\ level + \delta_8 Retired + \delta_9 Unable\ to\ work \\ & + \delta_{10} Not\ employed + \delta_{11} Make\ ends\ meet\ easily + \delta_{12} Northern + \delta_{13} Central \\ & + \delta_{14} Eastern + \delta_{15} Without\ partner + \delta_{16} Number\ of\ children + \delta_{17} Social\ network\ size \\ & + \delta_{18} Moderate\ physical\ activity\ 1/week + \delta_{19} Moderate\ physical\ activity\ 1 \\ & - 3\ per\ month + \delta_{20} Moderate\ physical\ activity\ hardly\ ever\ or\ never \\ & + \delta_{21} Number\ of\ ADL\ limitations + \delta_{22} Grip\ strength + \delta_{23} Cognitive\ function \\ & + \delta_{24} Depressive\ symptom + \zeta \end{aligned}$$

With $Z = \mathbf{I}(Z^* > 0)$, $\zeta \sim N(0,1)$, and $\mathbf{I}(\cdot)$ is the indicator function, when the covariates contain all of the confounders then $\varepsilon(0)$, $\varepsilon(1)$ are independent of ζ given the covariates.

A doubly robust estimator of τ is obtained by combining the fitted and predicted values for $Y_{i\,(1)}$, $Y_{i\,(0)}$, and Z_i for all individuals in the sample $i = 1,2,..,n$. See Genbäck and de Luna (2019) (Eq.(8)) for more details. In the same paper, a detailed description is given on how to obtain uncertainty intervals for τ that allow for unobserved confounders, i.e. for ρ = correlation (Ɛ(j), ζ) ≠ 0. The estimator and the uncertainty intervals are implemented in the package UI in R (available on CRAN, https://cran.r-project.org/package=ui) which we used in this study.

Table C1. Average causal effect (ACE) of receiving support, providing support, social participation on quality of life, on average, by education level

	ACE	95% Confidence Interval	95% Uncertainty Interval
**Providing support**			
Education level			
Low	**0.319**	**(0.006,0.632)**	(-0.412,1.051)
Middle	-0.004	(-0.19,0.182)	(-0.531,0.523)
High	0.101	(-0.085,0.287)	(-0.391,0.593)
**Receiving support**			
Education level			
Low	**-0.388**	**(-0.636,-0.14)**	(-1.073,0.298)
Middle	**-0.338**	**(-0.532,-0.144)**	(-0.911,0.235)
High	**-0.245**	**(-0.461,-0.029)**	(-0.796,0.306)
**Participation in social activity**			
Education level			
Low	**0.581**	**(0.341,0.821)**	(-0.058,1.221)
Middle	**0.496**	**(0.344,0.647)**	**(0.002,0.990)**
High	0.205	(0.022,0.389)	(-0.324,0.735)

References

Genbäck, M., & de Luna, X. (2019). Causal inference accounting for unobserved confounding after outcome regression and doubly robust estimation. *Biometrics, 75*(2), 506-515. https://doi.org/10.1111/biom.13001

D. Additional descriptive analyses

Table D1. Gender, age and region distribution by the main three predictors

	Participation in social activity				Providing social support				Receiving social support			
	No		Yes		No		Yes		No		Yes	
	n	%	n	%	n	%	n	%	n	%	n	%
Gender												
Woman	12223	57.1	9174	42.9	14937	69.8	6460	30.2	16287	76.1	5110	**23.9**
Man	8978	54.4	7533	**45.6**	11304	68.5	5207	**31.5**	13535	**82.0**	2976	18.0
Age group												
50-64	8054	51.6	7561	**48.4**	9591	61.4	6024	**38.6**	12864	**82.4**	2751	17.6
65-74	7382	54.4	6185	45.6	9469	69.8	4098	30.2	10855	80.0	2712	20.0
75+	5765	66.1	2961	33.9	7181	82.3	1545	17.7	6103	69.9	2623	**30.1**
Region												
Southern	6263	76.7	1898	23.3	6921	84.8	1240	15.2	7144	**87.5**	1017	12.5
Central	5931	43.4	7743	56.6	8739	63.9	4935	36.1	10873	79.5	2801	20.5
Northern	1372	27.2	3675	**72.8**	2661	52.7	2386	**47.3**	3807	75.4	1240	24.6
Eastern	7635	69.2	3391	30.8	7920	71.8	3106	28.2	7998	72.5	3028	**27.5**
Providing social support												
No	16563	63.1	9678	36.9					21634	**82.4**	4607	17.6
Yes	4638	39.8	7029	**60.2**					8188	70.2	3479	**29.8**
Receiving social support												
No	16782	56.3	13040	43.7	21634	72.5	8188	27.5				
Yes	4419	54.7	3667	**45.3**	4607	57	3479	**43.0**				
Participation in social activity												
No					16563	78.1	4638	21.9	16782	**79.2**	4419	20.8
Yes					9678	57.9	7029	**42.1**	13040	78.1	3667	**21.9**
